# An Update on 2,5-Diketopiperazines from Marine Organisms

**DOI:** 10.3390/md12126213

**Published:** 2014-12-19

**Authors:** Ri-Ming Huang, Xiang-Xi Yi, Yuying Zhou, Xiangdong Su, Yan Peng, Cheng-Hai Gao

**Affiliations:** 1 Key Laboratory of Plant Resources Conservation and Sustainable Utilization, South China Botanical Garden, Chinese Academy of Sciences, Guangzhou 510650, China; E-Mail: huangriming@scib.ac.cn; 2 Department of Pharmacy and Pharmacology, University of Bath, Bath BA2 7AY, UK; E-Mail: prsxs@bath.ac.uk; 3 School of Pharmaceutical Sciences, Guangxi University of Chinese Medicine, Nanning 530001, China; E-Mail: xiangxiyi81@aliyun.com; 4 Department of Cell Biology, Jinan University, Guangzhou 510632, China; E-Mail: chenlu0268 @163.com; 5 Life Science & Technology School, Lingnan Normal University, Zhanjiang 52048, China; E-Mail: py00_2006@126.com; 6 Key Laboratory of Marine Environmental Science, Guangxi Academy of Sciences, Nanning 530007, China

**Keywords:** 2,5-diketopiperazine, marine organism, biosynthetic processes, biological activity

## Abstract

2,5-Diketopiperazines (2,5-DKPs) are an important category of structurally diverse cyclic dipeptides with prominent biological properties. These 2,5-DKPs have been obtained from a variety of natural resources, including marine organisms. Because of the increasing numbers and biological importance of these compounds, this review covers 90 marine originated 2,5-DKPs that were reported from 2009 to the first half-year of 2014. The review will focus on the structure characterizations, biological properties and proposed biosynthetic processes of these compounds.

## 1. Introduction

2,5-Diketopiperazines (2,5-DKPs) are important cyclodipeptides derived from the “head to tail” cyclization of two α-amino acids. These molecules with the double lactam core structure of 2,5-DKPs, have previously been isolated from a variety of natural resources, including marine organisms. These small, conformationally rigid, chiral templates have multiple sites in 2,5-DKPs for the structural elaboration of diverse functional groups with defined stereochemistry. These characteristics not only enable them to show a broad range of biological activities [1], but also allow the development of the drug-like physicochemical properties. The structures, reactions, medicinal chemical properties and potential therapeutic applications of 2,5-DKPs, particularly that with the interesting biological activities have previously been reviewed [2,3]. However, 2,5-DKPs belong to a relatively unexplored category of the bioactive cyclic peptides that may hold a great promise for the potential medicinal use in the future. Our previous review [1] focused on the marine-derived 2,5-DKPs, covering their structures, names, biological studies and proposed biosynthetic process. This review aims to summarize 90 marine organisms-derived 2,5-DKPs published from 2009 to the first half year of 2014. This update is taxonomically presented based on the origin of the isolation of these 2,5-DKPs.

## 2. Marine Microorganisms

### 2.1. Actinomycetes

**Figure 1:**
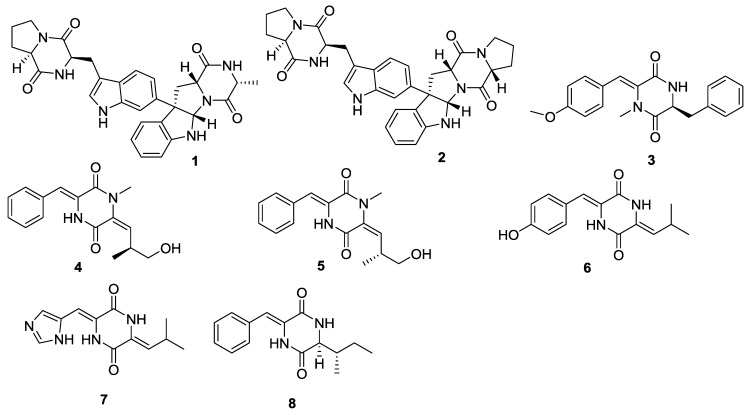
Structures of 2,5-DKPs from marine-derived actinomycetes.

**Scheme 1:**
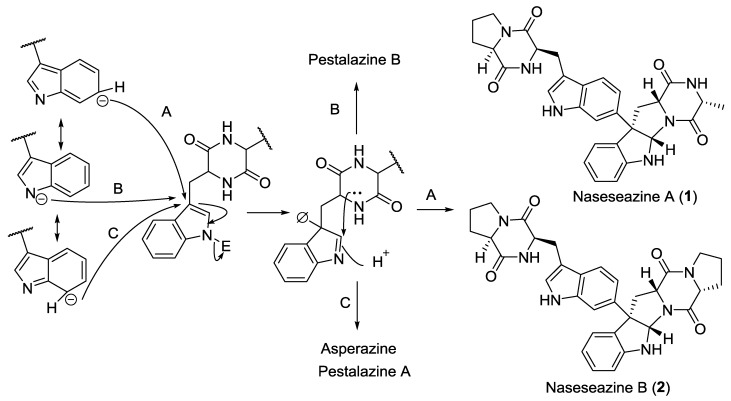
A plausible biosynthetic route to naseseazines A (**1**) and (**2**) and other related DKPs [4].

Eight 2,5-DKPs (**1**–**8**) have been obtained from actinomycetes (Figure 1 and Table 1). Naseseazines A (**1**) and B (**2**), with a new dimeric structural backbone, were isolated from the culture of *Streptomyces* sp. (sediment, Fiji), and a plausible biosynthetic route to the naseseazines and other related dimeric DKPs was also proposed (Scheme 1) [4]. The DKP derivative, nocazine C (**3**) was isolated from the actinomycete *Nocardiopsis dassonvillei* (sediment, Yellow River estuary, Dongying, China) [5]. The structure of nocazine C (**3**) had previously been reported [6], however, no experimental data were released to support the proposal structure [5]. In addition, five more DKP derivatives, (3*Z*,6*E*)-1-*N*-methyl-3-benzylidene-6-(2*S*-methyl-3-hydroxypropylidene)piperazine-2,5-dione (**4**), (3*Z*,6*E*)-1-*N*-methyl-3-benzylidene-6-(2*R*-methyl-3-hydroxypropylidene)piperazine-2,5-dione (**5**), (3*Z*,6*Z*)-3-(4-hydroxybenzylidene)-6-isobutylidenepiperazine-2,5-dione (**6**), (3*Z*,6*Z*)-3-((1*H*-imidazol-5-yl)-methylene)-6-isobutylidenepiperazine-2,5-dione (**7**) and (3*Z*,6*S*)-3-benzylidene-6-(2*S*-but-2-yl)piperazine-2,5-dione (**8**) were isolated from the actinomycete *Streptomyces* sp. FXJ7.328 (sediment, Huanghai beach, China) [7].

### 2.2. Bacteria

**Figure 2:**
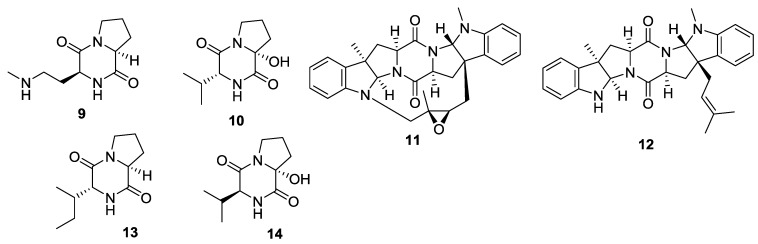
Structures of 2,5-DKPs from marine-derived bacteria.

Six 2,5-DKPs (**9**–**14**) have been obtained from marine-derived bacteria (Figure 2 and Table 2). Both bacillusamides A (**9**) and B (**10**) were isolated from the *Bacillus* sp. (sea urchin *Anthocidaris crassispina*, Nagasaki Shitsu coast, Japan). It was reported that bacillusamide A (**9**) exhibited a modest inhibitory property against *Aspergillus niger* [8]. The prenylated DKPs, norcardioazines A (**11**) and B (**12**) were obtained from the *Nocardiopsis* sp. (sediment, South Molle, Is., Brisbane, Australia), of which norcardioazine A (**11**) was found to be a noncytotoxic MDR reversing agent [9]. Furthermore, two DKP derivatives, staphyloamides A (**13**) and B (**14**) were isolated from the culture broth of *Staphylococcus* sp. (algae *Corallina officinalis* Lineaus, Nagasaki Shitsu coast of Japan) [10].

**Table 1:** 2,5-DKPs from marine-derived actinomycetes.

Number	Name	Bioactivity	Source	Reference(s)
**1**	Naseseazine A	-	*Streptomyces* sp.	[4]
**2**	Naseseazine B	-	*Streptomyces* sp.	[4]
**3**	Nocazine C	-	*Nocardiopsis dassonvillei*	[5,6]
**4**	(3 *Z*,6*E*)-1-*N*-methyl-3-benzylidene-6-(2*S*-methyl-3-hydroxypropylidene)piperazine-2,5-dione	-	*Streptomyces* sp.	[7]
**5**	(3 *Z*,6*E*)-1-*N*-methyl-3-benzylidene-6-(2*R*-methyl-3-hydroxypropylidene)piperazine-2,5-dione	-	*Streptomyces* sp.	[7]
**6**	(3 *Z*,6*Z*)-3-(4-hydroxybenzylidene)-6-isobutylidenepiperazine-2,5-dione	Modest antivirus activity against influenza A (H1N1) virus	*Streptomyces* sp.	[7]
**7**	(3 *Z*,6*Z*)-3-((1*H*-imidazol-5-yl)-methylene)-6-isobutylidenepiperazine-2,5-dione (7)	-	*Streptomyces* sp.	[7]
**8**	(3 *Z*,6*S*)-3-benzylidene-6-(2*S*-but-2-yl)piperazine-2,5-dione	-	*Streptomyces* sp.	[7]

**Table 2:** 2,5-DKPs from marine-derived bacteria.

Number	Name	Bioactivity	Source	Reference
**9**	Bacillusamide A	Weak inhibition activity against *A. niger*	*Bacillus* sp.	[8]
**10**	Bacillusamide B	-	*Bacillus* sp.	[8]
**11**	Norcardioazine A	Inhibition of *P*-Glycoprotein	*Nocardiopsis* sp.	[9]
**12**	Norcardioazine B	-	*Nocardiopsis* sp.	[9]
**13**	Staphyloamide A	-	*Staphylococcus* sp.	[10]
**14**	Staphyloamide B	-	*Staphylococcus* sp.	[10]

### 2.3. Fungi

**Figure 3:**
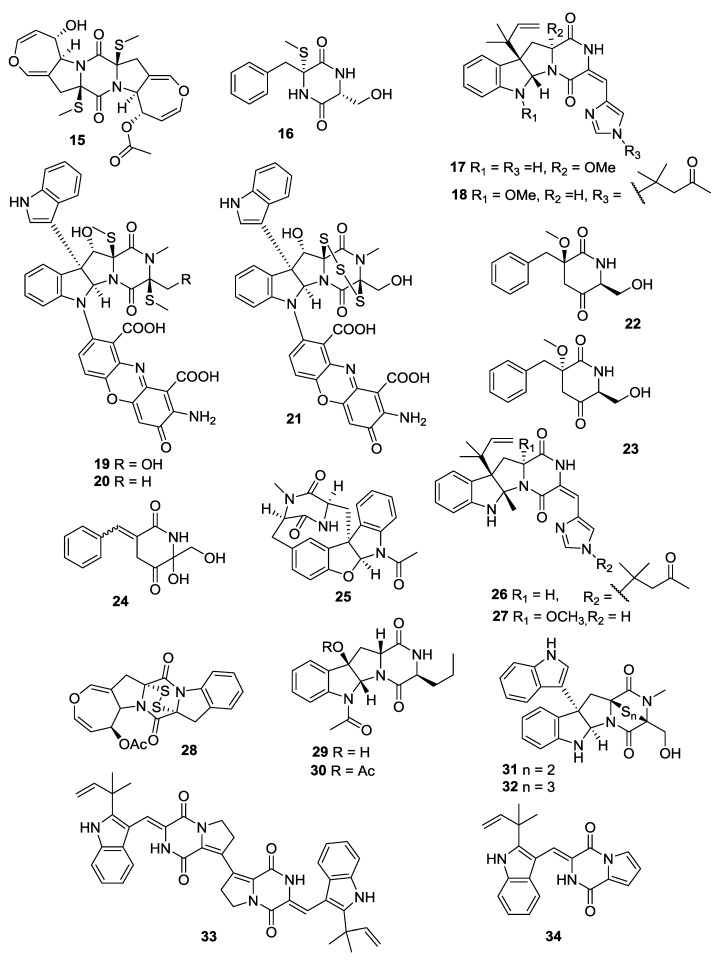

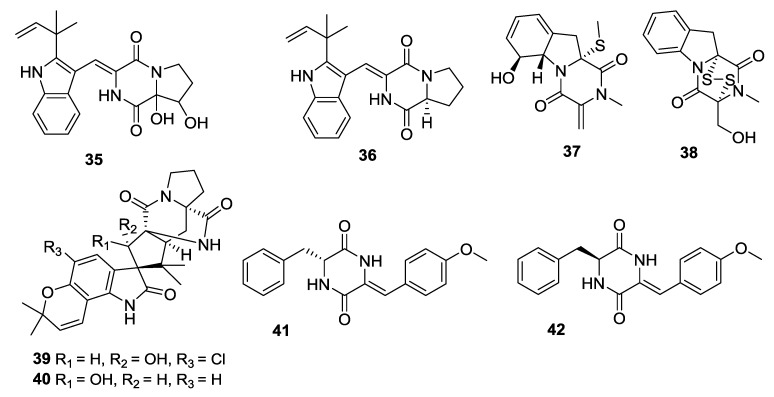
Structures of 2,5-DKPs from marine-derived fungi of sediment origin.

**Figure 4:**
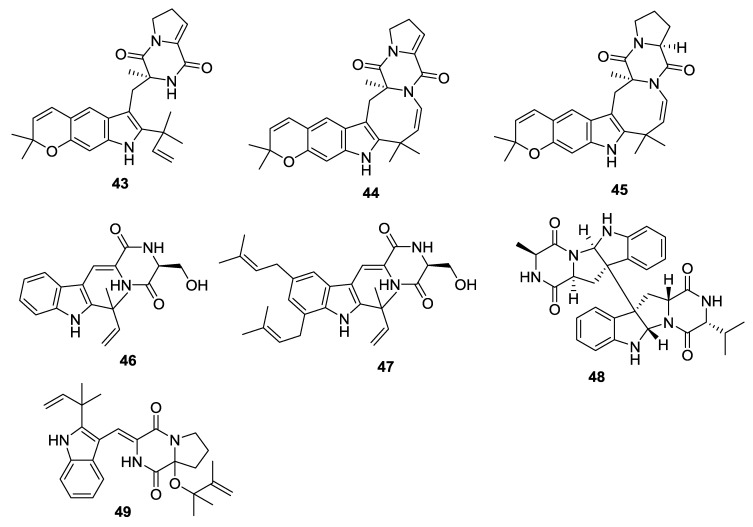
Structures of 2,5-DKPs from marine-derived fungi of algae origin.

**Figure 5:**
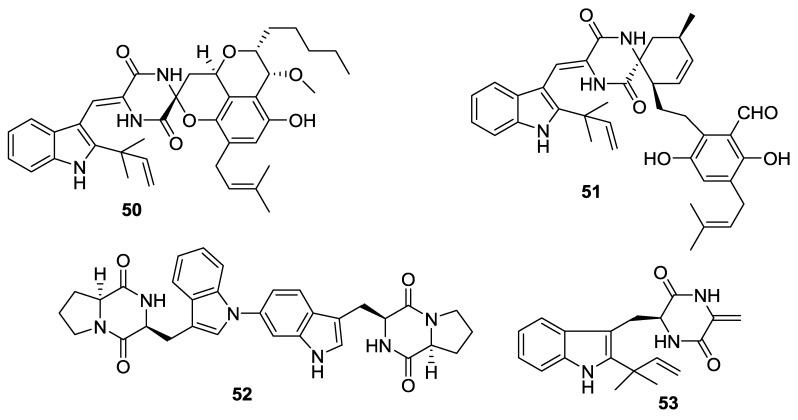
Structures of 2,5-DKPs from marine-derived fungi of mangrove rhizosphere soil origin.

**Figure 6:**
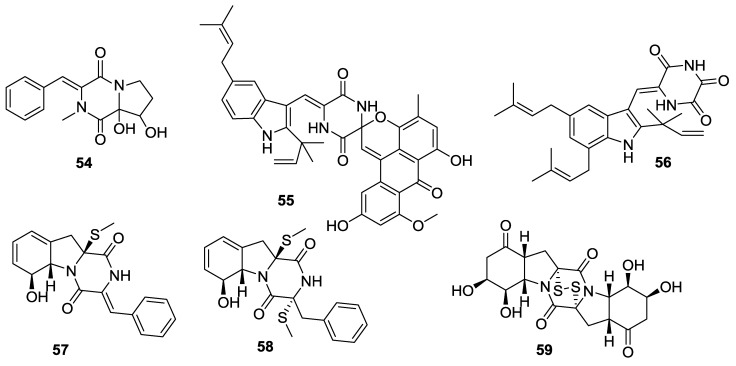
Structures of 2,5-DKPs from marine-derived fungi of mangrove origin.

**Figure 7:**
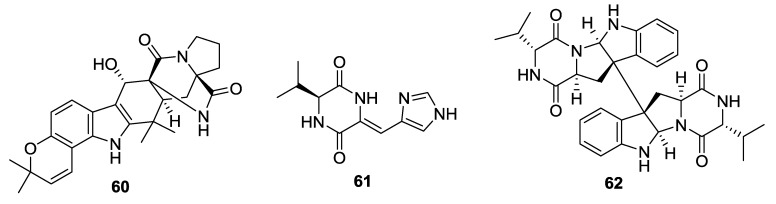
Structures of 2,5-DKPs from marine-derived fungi of sponge origin.

**Figure 8:**
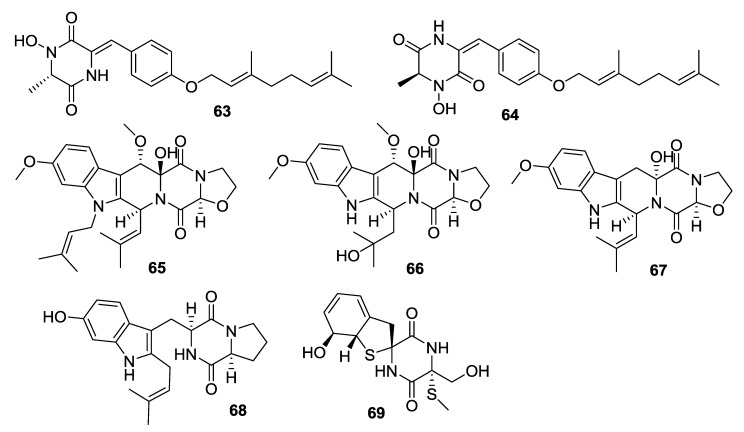
Structures of 2,5-DKPs from marine-derived fungi of mud origin.

**Figure 9:**
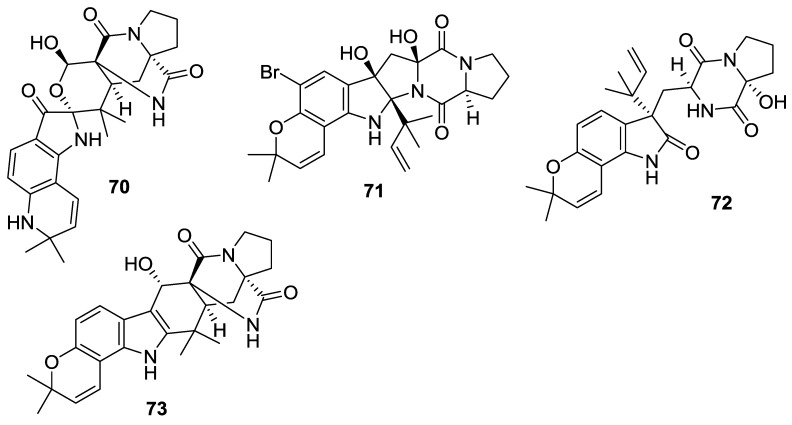
Structures of 2,5-DKPs from marine-derived fungi of mollusk origin.

**Figure 10:**
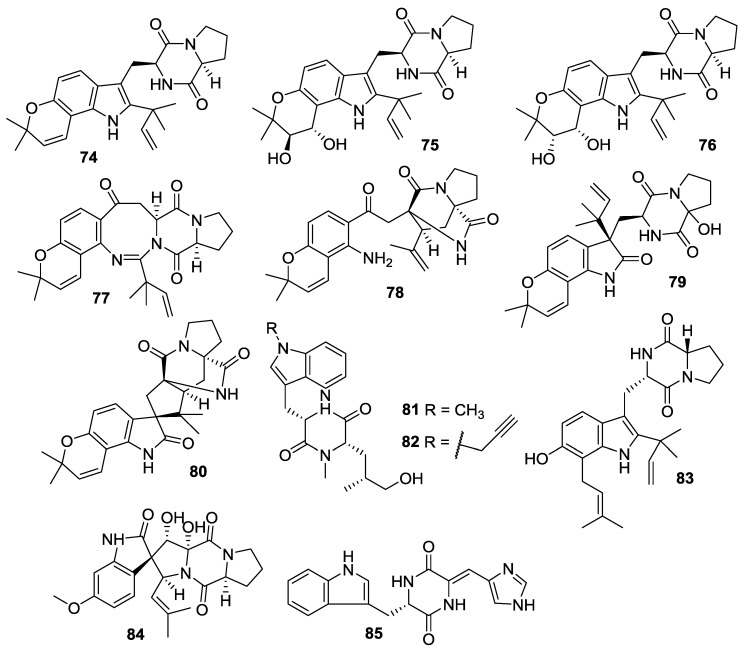
Structures of 2,5-DKPs from marine-derived fungi of other origins.

In the past five years, marine-derived fungi have been shown to be the rich sources of 2,5-DKP derivatives. Seventy-one 2,5-DKPs (**15**–**85**) have been obtained from the marine fungi (Figure 3, Figure 4, Figure 5, Figure 6, Figure 7, Figure 8, Figure 9 and Figure 10 and Table 3, Table 4, Table 5, Table 6, Table 7, Table 8, Table 9 and Table 10).

**Table 3:** 2,5-DKPs from marine-derived fungi of sediment origin.

Number	Name	Bioactivity	Source	Reference
**15**	Alternarosin A	-	*Alternaria raphani*	[11]
**16**	Not given	-	*fumigatus*	[12]
**17**	Roquefortine F	Cytotoxic activity	*Penicillium* sp.	[13]
**18**	Roquefortine G	Cytotoxic activity	*Penicillium* sp.	[13]
**19**	Plectoshpaeroic acid A	Inhibitor of indoleamine 2,3-dioxygenase (IDO)	*Plectosphaerella cucumerina*	[14]
**20**	Plectoshpaeroic acid B	Inhibitor of indoleamine 2,3-dioxygenase (IDO)	*P. cucumerina*	[14]
**21**	Plectoshpaeroic acid C	Inhibitor of indoleamine 2,3-dioxygenase (IDO)	*P.cucumerina*	[14]
**22**	Not given	-	*A*. *fumigatus*	[15]
**23**	Not given	-	*A*. *fumigatus*	[15]
**24**	Not given	-	*A*. *fumigatus*	[15]
**25**	Azonazine	Anti-inflammatory activity	*A*. *insulicola*	[16]
**26**	Roquefortine H	-	*Penicillium* sp.	[17]
**27**	Roquefortine I	-	*Penicillium* sp.	[17]
**28**	Deoxyapoaranotin	-	*A. versicolor*	[18]
**29**	Protuboxepin A	-	*Aspergillus* sp.	[19]
**30**	Protuboxepin B	-	*Aspergillus* sp.	[19]
**31**	Luteoalbusin A	Potent cytotoxins against several HTCLs	*A. luteoaltus*	[20]
**32**	Luteoalbusin B	Potent cytotoxins against several HTCLs	*A. luteoaltus*	[20]
**33**	Brevianamide S	Significant antibacterial activity against Bacille Calmette-Guerin (BCG)	*A. versicolor*	[21]
**34**	Brevianamide T	-	*A. versicolor*	[21]
**35**	Brevianamide U	-	*A. versicolor*	[21]
**36**	Brevianamide V	-	*A. versicolor*	[21]
**37**	Bis(dethio)-10a-methylthio-3a-deoxy-3,3a-didehydrogliotoxin	-	*Penicillium* sp.	[22]
**38**	6-Deoxy-5a,6-didehydrogliotoxin	-	*Penicillium* sp.	[22]
**39**	5-Chlorosclerotiamide	-	*A. westerdijkiae*	[23]
**40**	10- *Epi*-sclerotiamide	-	*A. westerdijkiae*	[23]
**41**	Nocazine D	-	*Nocardiopsis alba*	[24]
**42**	Nocazine E	-	*N. alba*	[24]

**Table 4:** 2,5-DKPs from marine-derived fungi of algae origin.

Number	Name	Bioactivity	Source	Reference
**43**	Carneamide A	-	*A. carneus*	[25]
**44**	Carneamide B	-	*A. carneus*	[25]
**45**	Carneamide C	-	*A. carneus*	[25]
**46**	Cristatumin A	Moderate activity against *E. coli*	*E. cristatum*	[26]
**47**	Cristatumin B	Moderate lethal activity against brine shrimp	*E. cristatum*	[26]
**48**	Cristatumin C	-	*E.cristatum*	[26]
**49**	9Ɛ- *O*-2(2,3-dimethylbut-3-enyl)brevianamide Q	-	*A. versicolor*	[27]

**Table 5:** 2,5-DKPs from marine-derived fungi of mangrove rhizosphere soil origin.

Number	Name	Bioactivity	Source	Reference
**50**	Effusin A	-	*A. effuses*	[28]
**51**	Dihydrocryptoechinuline D	Potent activity on P388 cells with an IC_50_ value of 1.83 μM	*A. effuses*	[28]
**52**	Aspergilazine A	Weak activity against influenza A (H1N1) virus	*A. taichungensis*	[29]
**53**	Dihydroneochinulin B	Weak activity against BEL-7402 and A-549 cell lines	*A. effuses*	[30]

**Table 6:** 2,5-DKPs from marine-derived fungi of mangrove origin.

Number	Name	Bioactivity	Source	Reference
**54**	3-Benzylidene-8,8a-dihydroxy-2-methyl-hexahydro-pyrrolo[1,2-a]pyrazine-1,4-dione	-	unidentified	[31]
**55**	7- *O*-methylvariecoloride A	-	*E. rubrum*	[32]
**56**	12-Demethyl-12-oxo-eurotechinulin B	Displayed cytotoxic activities	*E. rubrum*	[33]
**57**	Phomazine A	-	*Phoma* sp.	[34]
**58**	Phomazine B	Moderate cytotoxicities against the HL-60, HCT-116, K562, MGC-803 and A549 cell lines	*Phoma* sp.	[34]
**59**	Phomazine C	-	*Phoma* sp.	[34]

**Table 7:** 2,5-DKPs from marine-derived fungi of sponge origin.

Number	Name	Bioactivity	Source	Reference
**60**	21-Hydroxystephacidin	-	*A. ostianus*	[35]
**61**	Pre-aurantiamine	-	*A. aculeatus*	[36]
**62**	Eurocristatine	-	*E. cristatum*	[37]

**Table 8:** 2,5-DKPs from marine-derived fungi of mud origin.

Number	Name	Bioactivity	Source	Reference
**63**	Gliocladride A	Cytotoxic activity against HL-60, U937 and T47D with IC_50_ values form 12.80 μg/mL to 42.80 μg/mL	*Gliocldium* sp.	[38]
**64**	Gliocladride B	Cytotoxic activity against HL-60, U937 and T47D with IC_50_ values form 11.60 μg/mL to 52.83 μg/mL	*Gliocldium* sp.	[38]
**65**	Prenylcyclotryprostatin B	Most potent activities against both U937 and PC-3 cell lines	*A. fumigatus*	[39]
**66**	20-Hydroxycyclotryprostatin B	Most potent activities against both U937 and PC-3 cell lines	*fumigatus,* *A. sydowii*	[39–41]
**67**	9-Hydroxyfumitremorgin C	Most potent activities against both U937 and PC-3 cell lines	*A. fumigatus*	[39]
**68**	6-Hydroxytryprostatin B	Most potent activities against both U937 and PC-3 cell lines	*A. fumigatus*	[39]
**69**	Spirogliotoxin	Most potent activities against both U937 and PC-3 cell lines	*A. fumigatus*	[39]

**Table 9:** 2,5-DKPs from marine-derived fungi of mollusk origin.

Number	Name	Bioactivity	Source	Reference
**70**	Notoamide O	-	*Aspergillus* sp.	[42]
**71**	Notoamide P	-	*Aspergillus* sp.	[42]
**72**	Notoamide Q	-	*Aspergillus* sp.	[42]
**73**	Notoamide R	-	*Aspergillus* sp.	[42]

**Table 10:** 2,5-DKPs from marine-derived fungi of other origins.

Number	Name	Bioactivity	Source	Reference
**74**	Notamide E	-	*Aspergillus* sp.	[43,44]
**75**	Notamide E2	-	*Aspergillus* sp.	[45]
**76**	Notamide E3	-	*Aspergillus* sp.	[45]
**77**	Notamide E4	-	*Aspergillus* sp.	[45]
**78**	Notamide L	-	*Aspergillus* sp.	[46]
**79**	Notamide M	-	*Aspergillus* sp.	[46]
**80**	Notamide N	-	*Aspergillus* sp.	[46]
**81**	Cyclomarazine M	-	*Salinispora arenicola*	[47]
**82**	Cyclomarazine P	-	*S*. *arenicola*	[47]
**83**	Notoamide S	-	*Aspergillus* sp.	[48,49]
**84**	Spirotryprostatin F	Stimulatory phytoregulatory activity	*A. fumigatus*	[50]
**85**	Penilloid A	-	*Penicillium* sp.	[51]

#### 2.3.1. Fungi from Sediment Origin

**Scheme 2:**
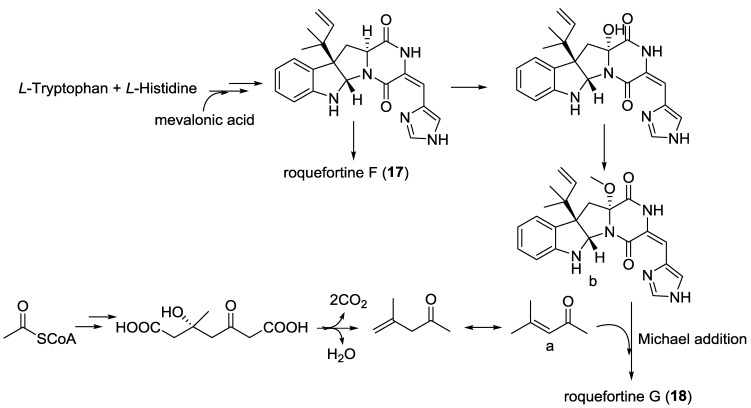
Postulated biosynthetic pathway for **17** and **18** [13].

An *Alternaria raphani* (sediment, sea salt field, Qingdao, China) yielded a DKP alkaloid, alternarosin A (**15**) [11]. A gliotoxin analogue (**16**) was isolated from the *Aspergillus fumigatus* (sediment, Jiaozhou Bay, Qingdao, China) [12], and the configuration assignment of **16** was then revised [12]. The DKP alkaloids, roquefortines F (**17**) and G (**18**) were isolated from the *Penicillium* sp. (sediment, 5080 m, location not given), and their biogenetic relationships were postulated in a plausible pathway (Scheme 2) [13]. The DKP alkaloids, plectoshpaeroic acids A–C (**19**–**21**) were isolated from the *Plectosphaerella cucumerina* (sediments, Barkley Sound, British Columbia) and identified as inhibitors of indoleamine 2,3-dioxygenase (IDO), an enzyme catalyzing the conversion of the essential amino acid l-tryptophan to *N*-formylkynurenine [14]. DKPs (**22**–**24**) were isolated from the *A*. *fumigatus* (sediment, Jiaozhou Bay, China) [15]. The fermentation of *A*. *insulicola* (sediment, Hawaii) resulted in the discovery of a hexacyclic dipeptide azonazine (**25**), which exhibited an anti-inflammatory activity through the inhibition of NF-κB luciferase and nitrite production [16]. Two DKP derivatives, roquefortines H (**26**) and I (**27**) were isolated from the *Penicillium* sp. (deep ocean sediment, unspecified location) [17]. The DKP disulfide deoxyapoaranotin (**28**) from the *A. versicolor* (sediment, East Sea, Korea) was reported to be cytotoxic, and it showed an induced apoptotic activity against HCT-116 [18]. An *Aspergillus* sp. (sediment, Dadaepo Beach, Busan, Korea) gave two DKP-type alkaloids (**29**) and (**30**) [19]. Two indole DKPs, luteoalbusins A (**31**) and B (**32**) were isolated from the *Acrostalagmus luteoaltus* (deep sea sediment, South China Sea), and they were identified as potent cytotoxins against several HTCLs [20]. The dimeric DKP brevianamide S (**33**) and the monomeric brevianamides T–V (**34**–**36**) were isolated from the *A. versicolor* (sediment, Bohai Sea, China). A plausible biosynthetic relationship linking these brevianamide derivatives through a sequence of oxidative transformations was described. Brevianamide S (**33**) showed a selective activity against the Bacille Calmette-Guérin (BCG) strain of *Mycobacterium bovis*, which suggested a new mechanism of the action with potential to be an antitubercular drug lead [21]. The isolation of the *Penicillium* sp. (deep sea sediment, Suruga Bay, Japan) yielded two gliotoxin-related compounds, including **37** and **38**. Compound (**38**) was shown to be cytotoxic to P388 cells [22]. Two prenylated indole alkaloids, 5-chlorosclerotiamide (**39**) and 10-*epi*-sclerotiamide (**40**) were isolated from the deep-sea-derived fungus *A. westerdijkiae* DFFSCS013 (sediment, South China Sea) [23]. Two DKP enantiomers, nocazines D (**41**) and E (**42**) were isolated from the *Nocardiopsis alba* SCSIO 03039 (sediment, Indian Ocean) [24].

#### 2.3.2. Fungi from Algae Origin

**Scheme 3:**
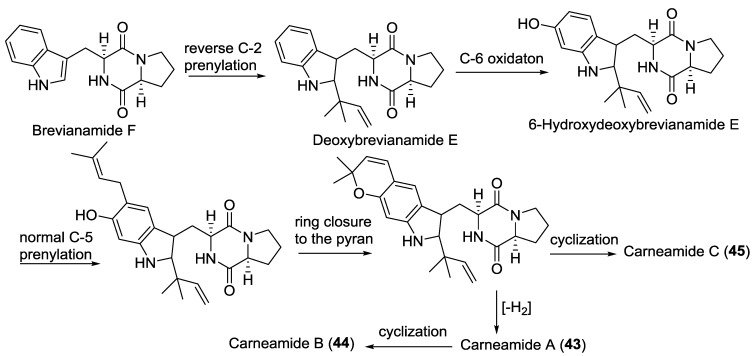
Postulated biosynthetic pathway for carneamides A–C (**43**–**45**) [25].

An *A. carneus* (brown alga *Laminaria sachalinensis*, Kunachir Is., Russia) was the source of the prenylated indole alkaloids, carneamides A–C (**43**–**45**), for which a possible biosynthetic pathway was proposed (Scheme 3) [25]. The endophytic fungus *Eurotium cristatum* (brown alga *Sargassum* *thunbergii*, location unspecified) was the source of the indole alkaloids, cristatumins A–C (**46**–**48**) [26]. 9Ɛ-*O*-2(2,3-dimethylbut-3-enyl)brevianamide Q (**49**) was isolated from the endophytic fungus *A. versicolor* (brown alga *S. thunbergii*, Pingtan Is., China) [27].

#### 2.3.3. Fungi from Mangrove Rhizosphere Soil Origin

The racemic spiroalkaloids, effusin A and dihydrocryptoechinulin D (shown here as one of the enantiomers, effusin A (**50**) and dihydrocryptoechinuline D (**51**), respectively) were obtained from the *A. effuses* (mangrove rhizosphere soil, Fujian, China). The racemates were subsequently resolved, and their absolute configurations were determined by the solution time dependent density function theory (TDDFT) and electronic CD (ECD) calculations. Effusin A (**50**) contains a spirobicyclic *N*,*O*-acetal moiety, which could be obtained by a domino ring-closure reaction between the substituted salicylaldehyde moiety in aspergin and the eneamide moiety of the DKP unit in neoechinulin B. In contrast, an enzyme-catalyzed regiospecific [4 + 2] Diels-Alder reaction produces the spirobicycle of dihydrocryptoechinuline D (**51**). The racemate of dihydrocryptoechinulin D inhibited the growth of P388 cells, and the (12*R*,28*S*,31*S*)-enantiomer 51 showed a selective, moderate inhibition of topoisomerase I [28]. Aspergilazine A (52), a DKP dimer consisting of two DKP units with a rare N-1 to C-6 linkage, was obtained from the *A. taichungensis* (mangrove root soil, *Acrostichum aureum*, source not given) [29]. The prenylated indole DKP alkaloid, dihydroneochinulin B (**53**) was isolated from the fungus *A. effuses* H1-1 (mangrove rhizosphere soil, Fujian, China) [30].

#### 2.3.4. Fungi from Mangrove Origin

The amide alkaloid (**54**) was isolated from an unidentified endophytic fungus (mangrove, *Acanthus ilicifolius*, South China Sea) [31]. The spirocyclic DKP alkaloid, 7-*O*-methylvariecoloride A (**55**) was sourced from the *Eurotium rubrum* (stem of the mangrove *Hibiscus tiliaceus*, Hainan Is., China) [32]. An endophytic strain of *E. rubrum*, (semi-mangrove *H. tiliaceus*, Hainan Is., China) produced a DKP alkaloid, 12-demethyl-12-oxo-eurotechinulin B (**56**) [33]. Furthermore, three thiodiketopiperazines, phomazines A–C (**57**–**59**) were isolated from an endophytic fungus *Phoma* sp. OUCMDZ-1847 (mangrove *Kandelia candel*, Wenchang, Hainan Province, China) [34].

#### 2.3.5. Fungi from Sponge Origin

A heptacyclic alkaloid, 21-hydroxystephacidin (**60**) was isolated from the *A. ostianus* (unidentified sponge, Pohnpei) [35]. Pre-aurantiamine (**61**) was isolated from the *A. aculeatus* (sponge *Stylissa flabelliformis*, Phi Phi Is., Krabi Province, Thailand) [36]. The DKP dimer, eurocristatine (**70**) was isolated from the endophytic fungus *Eurotium cristatum* (sponge *Mycale* sp., Wonnapa Beach, Bangsaen, Thailand) [37].

#### 2.3.6. Fungi from Mud Origin

Two piperazine-2,5-dione derivatives, gliocladrides A (**63**) and B (**64**) were isolated from the *Gliocldium* sp. (sea mud, Rushan, China) [38]. Five DKPs, prenylcyclotryprostatin B (**65**), 20-hydroxycyclotryprostatin B (**66**), 9-hydroxyfumitremorgin C (**67**), 6-hydroxytryprostatin B (**68**) and spirogliotoxin (**69**) were isolated from the *A. fumigatus* (intertidal mud, Yingkou, China). Prenylcyclotryprostatin B and 9-hydroxyfumitremorgin C were shown to be moderate inhibitors of human leukemic monocyte lymphoma (U937) cells [39]. 20-Hydroxycyclotryprostatin B was also isolated from two other sources in 2012, firstly from the *A.* *sydowii* (gorgonian coral *Verrucella umbraculum*, Sanya, Hainan province, China) as cyclotryprostatin E [40] and secondly, from a terrestrial *A. fumigatus* as 12β-hydroxy-13α-methoxyverruculogen TR-2 [41].

#### 2.3.7. Fungi from Mollusk Origin

**Scheme 4:**
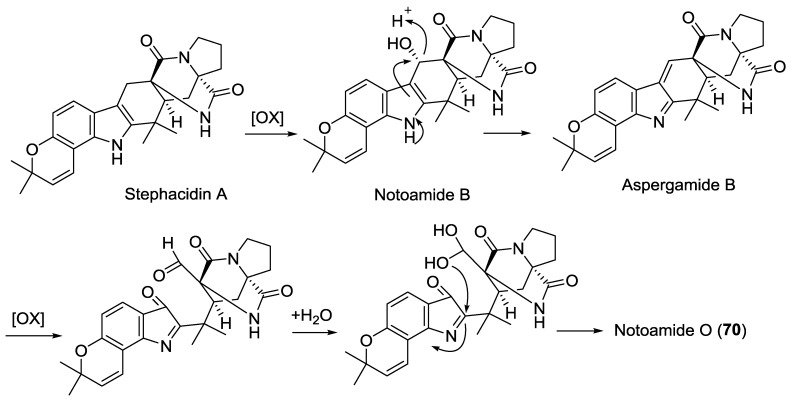
Postulated biosynthetic pathway for **70** [42].

The fermentation of *Aspergillus* sp. (mollusk *Mytilis edulis galloprovincialis*, Noto Penninsula, Sea of Japan) yielded the notoamides O–R (**70**–**73**). Notoamide O (**70**) is noteworthy as the compound consists of a novel hemiacetal/hemiaminal ether moiety, which represents an unusual branch point for the oxidative modification of other members in the family of the prenylated indole alkaloids in the biogenetic pathway (Scheme 4) [42]. The structure of notoamide Q (**72**) has been revised [42]. The whole genome sequencing of *Aspergillus* MF 297-2 (mollusk *M. edulis galloprovincialis*, Noto Penninsula, Japan Sea) [52] led to the identification and characterization of the biosynthetic gene cluster for stephacidins [53] and the notoamide alkaloids [43,52].

#### 2.3.8. Fungi from Other Origins

Notamide E (**74**) was isolated from the culture of *Aspergillus* sp. (mussel *Mytilus edulis*, Noto Peninsula, Sea of Japan) [43]. The compound (**74**) has been synthesized prior to its isolation from the natural source, and it was proposed [44] to be an advanced precursor to notoamides A–D. The biosynthetic studies of the producing organism indicated that notoamide E (**74**) was a short-lived metabolite. The feeding experiments utilizing synthetic, ^13^C-labelled (**74**) demonstrated the incorporation of notoamide E into notoamides C [43], D [45] and 3-*epi*-notamide C [44]. These studies also produced three minor alkaloids, notamides E2–E4 (**75**–**77**) [45]. Further investigation of the same culture of *Aspergillus* that yielded notamides A–D [45] led to the isolations of notamides L–N (**78**–**80**) [46]. In a direct and targeted gene manipulation experiment, the provision of synthetic *N*-alkyl tryptophan to a prenyltransferase-deficient mutant of a cyclomarin/cyclomarazine-producing *S*. *arenicola* led to the discovery of some novel derivatives, cyclomarazines M (**81**) and P (**82**) [47]. Two central pathway enzymes, which catalyzed both the normal and reverse prenyltransfer reactions, were characterized. The study also established the early steps of the biosynthetic procedure of prenylated indole alkaloid structure, including the production of notoamide S (**83**) [48]. Notoamide S (**83**) has been synthesized *via* *N*-Fmoc proline coupling with a 6-hydroxy-7-prenyl-2-reverse prenyl tryptophan derivative [49]. The spirocyclic DKP alkaloid, spirotryprostatin F (**84**) was isolated from the *A. fumigatus* (soft coral *Sinularia* sp., Kunashir Is., Kuril Islands), and it showed a stimulatory phytoregulatory activity in the study [50]. An indolyl DKP compound, penilloid A (**85**) was isolated from two marine derived fungi *Penicillium* sp. and *A.* *sydowii* [51]. 

## 3. Sponges

**Figure 11:**
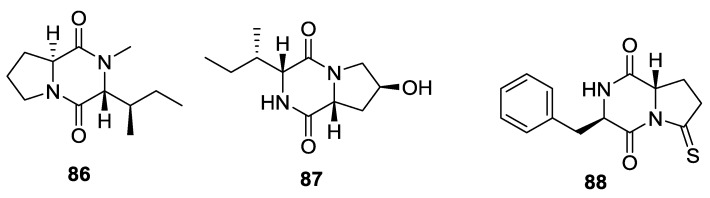
Structures of 2,5-DKPs from marine sponges.

Three 2,5-DKPs (**86**–**88**) have been isolated from sponges (Figure 11). A *Callyspongia* species (Haina Is., China) yielded callyspongidipeptide A (**86**) [54]. DKP 87 was obtained from *Stelletta* sp. (Jamieson Reef, Bonaparte Archipelago, Australia), and it was proposed to be the product of an enzymatically controlled condensation reaction between d-isoleucine and 4-*S*-hydroxy-d-proline [55]. A *Callyspongia* sp. (Haina Is., China) yielded callysponine A (**88**) [56].

## 4. Gorgonian

**Figure 12:**
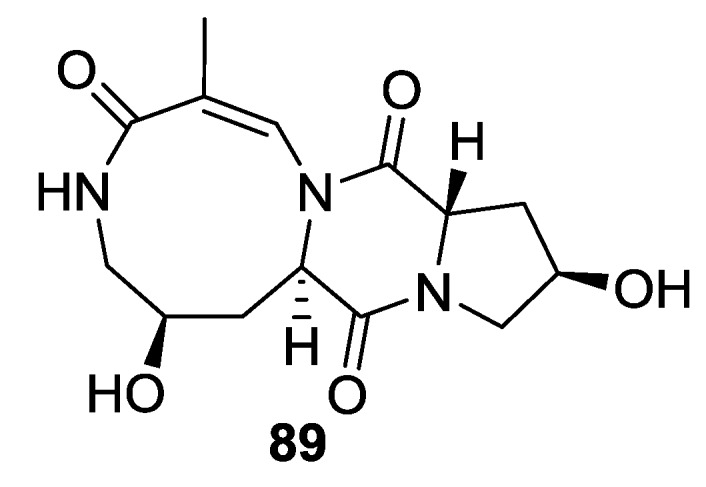
Structure of 2,5-DKP from gorgonian.

The isolation of the gorgonian *Menella kanisa* collected from Beibu Gulf led to the identification of menazepine A (**89**) (Figure 12) [57].

## 5. Red Algae

**Figure 13:**
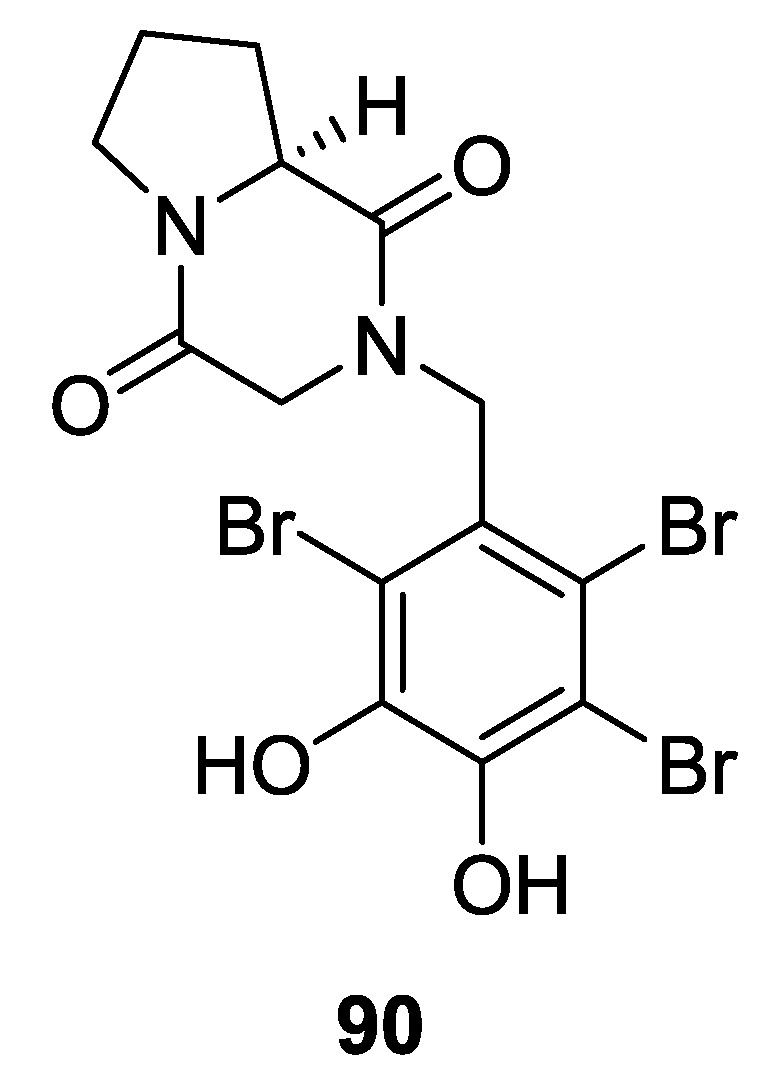
Structure of 2,5-DKP from red algae.

A collection of *Symphyocladia latiuscula* (Qingdao, Shandong Province, China) provided a bromophenol coupled to a DKP core structure (**90**) (Figure 13) [58].

## 6. Conclusions

**Figure 14:**
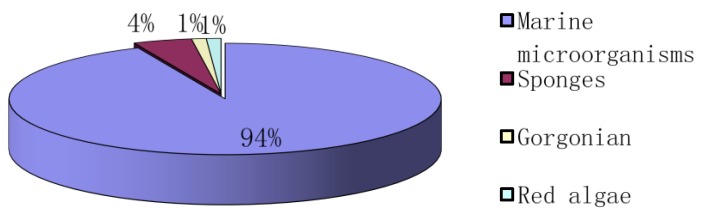
The distribution of 2,5-DKPs reported from marine organisms.

**Figure 15:**
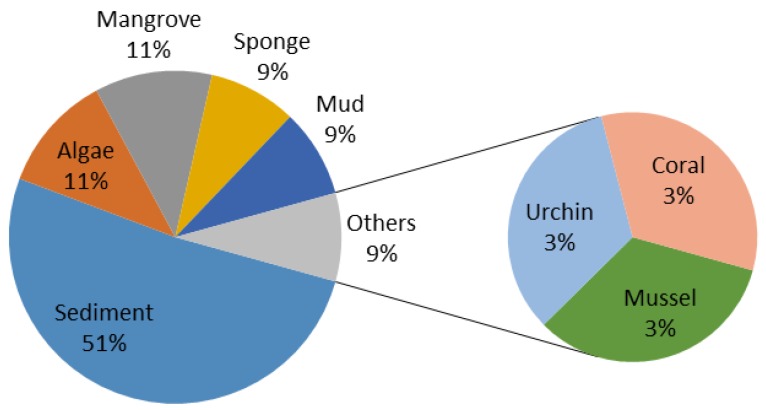
Origin of the microorganisms.

**Figure 16:**
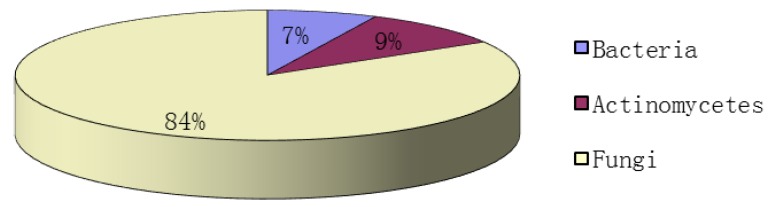
The distribution of 2,5-DKPs reported from marine-derived microorganisms.

2,5-DKPs are ubiquitous in nature. They have previously been isolated from bacteria, fungi, marine invertebrates and higher organisms [1,2]. Although these DPK derivatives have been known since the early 20th century, they only recently draw significant interests because of the diverse range of their biological activities [1], including the disruption of the biofilm formation through modulation of bacterial quorum sensing and their role in an interkingdom cell-cell signaling [42]. The increasing numbers of naturally occurring bioactive 2,5-DKPs have been obtained from various marine organisms, and the studies on these 2,5-DKPs have been the focus of many recent studies because of their potent biological activities. To date, more than 200 2,5-DKPs have been isolated from a diverse range of marine organisms, particularly marine microorganisms. Some of these 2,5-DKPs exhibited various bioactivities, such as cytotoxicity on cancer cell lines, anti-microbial and anti-inflammatory properties [1]. From 2009 to the first half-year of 2014, the main natural source of DKPs isolated from marine organisms is marine microorganisms, accounting for 94% (Figure 14). Many studies have performed on these bacteria, fungi and actinomycete that produced 2,5-DKPs, and these marine microorganisms were isolated from sediments, algae, mangrove, sponges and mud (Figure 15). It was indicated that 51% of the studied microorganisms were isolated from sediments as shown in Figure 15. Interestingly, the marine-derived fungi accounted for the largest part (84%) of total 2,5-DKPs that were isolated from marine microorganisms (Figure 16). The interest in natural products from marine microorganisms, especially marine-derived fungi, has increased significantly in the last decade [59,60], which has led to the discoveries of more 2,5-DKPs from the marine-derived fungi than that from other marine organisms. Consequently marine microorganisms, especially fungi can be a promising source of these bioactive 2,5-DKPs. The discovery of these compounds from marine-derived fungi demonstrates that some gene clusters in fungi may have the ability to produce structurally diverse DKPs by the biosynthetic pathways, which may need further investigations.
